# A Structural Bridge Between Kingdoms: How Collagen‐Derived Peptides Influence Plant Stress and Growth Pathways

**DOI:** 10.1111/ppl.70512

**Published:** 2025-09-14

**Authors:** Stefano Ambrosini, Alejandro Giorgetti, Marika Peli, Tiziana Pandolfini, Anita Zamboni, Zeno Varanini

**Affiliations:** ^1^ Department of Biotechnology University of Verona Verona Italy

**Keywords:** abiotic stress, collagen‐derived biostimulant, plant collagen‐binding proteins, root development

## Abstract

Collagen‐derived protein hydrolysates (CDPH) are widely used as plant biostimulants primarily due to their content of bioactive oligopeptides. When applied to hydroponically grown *Solanum lycopersicum* plants, CDPH significantly promoted root development, particularly by increasing the number and length of lateral roots. To gain insight into the underlying molecular mechanisms, we hypothesized that plants may possess proteins capable of interacting with collagen‐like peptides. To test this, we conducted a comprehensive homology search of the *Arabidopsis thaliana* proteome using a Hidden Markov model‐based approach built from three human collagen‐binding proteins (CBPs) and 14 known collagen‐binding domains (CBDs). After filtering, 10 *Arabidopsis* proteins emerged as putative candidates with the potential to bind collagen. Notably, the highest homology was observed for a matrix metalloproteinase, At5‐MMP, showing 44% identity with its human counterpart HsMMP1, and for AtSERPIN1, which displayed the strongest e‐value match to HsSERPINH1 (22% identity). Both plant proteins are functionally associated with responses to abiotic and biotic stresses, a feature that mirrors the known physiological effects of CDPH‐based biostimulants. These findings support the hypothesis that plants possess proteins capable of recognizing collagen‐like structures, offering a plausible molecular basis for the activity of CDPH‐based biostimulants and paving the way for future biochemical validation.

## 1 Introduction

Biostimulants represent a heterogeneous class of products capable of modulating plant physiological processes, offering promising avenues for enhancing growth, development, and stress resilience (du Jardin 2015). Among these, protein hydrolysates (PHs) are a prominent category (Moreno‐Hernández et al. 2022). Produced from the physical, chemical, or enzymatic breakdown of plant and animal by‐products and wastes, PHs are rich in oligopeptides and free amino acids (Cavani et al. 2003; Pituello et al. 2022). The beneficial effects of PHs on plants are well documented in the literature (Malécange et al. 2023). Among the animal‐derived PHs recognized as effective plant biostimulants are those obtained from keratin (chicken feathers), hemoglobin (erythrocytes), and collagen (bovine hair, epithelial tissues; also available as gelatin) (Cristiano et al. 2018; Tejada et al. 2018; Wilson et al. 2018). Although their plant growth‐promoting effects—such as enhanced crop growth, biomass accumulation, improved nutrient uptake, and greater abiotic stress tolerance—are well established, the molecular mechanisms underlying these effects remain largely unexplored. Moreover, the fundamental biological question of how plants perceive and respond to the bioactive molecules in these plant‐conditioning compounds remains unresolved. Notably, previous studies have demonstrated that a specific type of PH, collagen‐derived protein hydrolysate (CDPH), promotes maize root growth by modulating the expression of genes involved in cell wall organization, transport, stress response, and hormone metabolism (Ertani et al. 2013; Santi et al. 2017) and enhances tolerance to abiotic and nutritional stresses (Ambrosini et al. 2021). CDPH fractionation revealed a heterogeneous peptide composition (200–6500 Da) (Ambrosini et al. 2022) and circular dichroism analysis indicated that CDPH spectra closely match type II polyproline helices (PPII), suggesting the presence of peptides that retain this secondary structure (Ambrosini et al. 2021).

Collagens, the most abundant proteins in mammals, comprise approximately 30% of total protein mass and play critical roles in cellular and tissue development, including processes such as cell growth, differentiation, and migration (Ricard‐Blum 2011). The extracellular matrix contains 28 types of collagens, further diversified by isoforms and supramolecular organizations (Mienaltowski and Birk 2014). Other functional diversity arises from proteolytic cleavage, which releases bioactive peptides, and from cryptic functional sites exposed by conformational changes (Ricard‐Blum 2011). All collagens share at least one domain composed of three polypeptides folded into a triple helix, featuring a repetitive [GXY]_n_ motif, where X and Y are often proline (Pro) and 4‐hydroxyproline (Hyp) (Jariwala et al. 2022). Extensive research in humans has identified numerous collagen‐binding proteins (CBPs) and, in some cases, experimentally characterized collagen‐binding domains (CBDs). A review by Elango et al. (2022) highlights the remarkable diversity of proteins interacting with collagens, including receptor tyrosine kinases, integrins, immunoglobulin‐like receptors, and leukocyte receptor complexes (LRCs). Collagen fragments, from small fibrils to large triple‐helix segments, interact with receptors in various ways, eliciting distinct physiological responses. These findings suggest, as noted by Elango et al. (2022), an absence of a consistent molecular pattern in collagen‐mediated responses.

This study investigates whether collagen‐derived peptides can be recognized or bound by plant proteins. Specifically, we hypothesize that plants may possess putative collagen‐interacting proteins with structural similarities to known human collagen‐binding proteins (CBPs) and collagen‐binding domains (CBDs). Through computational analysis and predictive modeling, we aim to identify and characterize candidate collagen‐interacting proteins in two species chosen for their complementary value: *Arabidopsis thaliana* for its well‐annotated genome, facilitating in silico protein identification and structural prediction, and *Solanum lycopersicum* for its agronomic relevance and responsiveness to biostimulants.

## 2 Material and Methods

### 2.1 Hydroponics Growth and Biostimulant Treatment

*Solanum lycopersicum* seeds (Micro‐Tom) were sterilized and laid down in 8 g L^−1^ agar plates placed in the growth chamber to germinate for 5 days (120 μE m^−2^ s^−1^ average light intensity;16 h light/8 h dark photoperiod; 25°C). Similarly sized seedlings, with a root of approximately 3 cm, were transferred to 600‐mL pots containing a nutrient solution with the following composition: 400 μM CaSO_4_, 200 μM K_2_SO_4_, 200 μM KNO_3_, 175 μM KH_2_PO_4_, 100 μM MgSO_4_, 50 μM Na‐Fe‐EDTA, 25 μM (NH_4_)H_2_PO_4_, 5 μM KCl, 2.5 μM H_3_BO_3_, 0.2 μM MnSO_4_, 0.2 μM ZnSO_4_, 0.05 μM CuSO_4_, 0.05 μM (NH_4_)_6_Mo_7_O_24_, pH 6.0. Each pot accommodated 20 seedlings. Seedlings were treated with the CDPH at a concentration of 1.4 mg N L^−1^, or with an equivalent amount of N supplied as (NH_4_)H_2_PO_4_. The CDPH employed has been thoroughly characterized in previous publications (Santi et al. 2017; Ambrosini et al. 2021). The treatments were applied when plants were transferred to the hydroponic system (*t*_0_), 5 days after *t*_0_, and 7 days after *t*_0_. Plants were sampled an hour after the last treatment. Root parameters were analyzed with WinRHIZO software (EPSON V850 Pro, WinRHIZO‐ Pro2021a‐ Regent Instruments Inc.). Data were analyzed applying a two‐tailed Welch's *t*‐test (*p* > 0.05).

### 2.2 Protein Homology Assessment

The software HHPred (Meier and Söding 2015) was employed to assess the homology between human CBPs or CBDs and *Arabidopsis thaliana* proteins (default parameters). In particular, the Hidden Markov Models‐based program, HHsearch, within HHpred was used with default parameters. Here, we list the PDB IDs of the analyzed entries and the scientific work that determined the functional CBD, if present; otherwise, the CBP is indicated: Osteoclast associated immunoglobulin‐like receptor (OSCAR), 5CJB_1 (Haywood et al. 2016); Platelet glycoprotein VI (GP6), 2GI7_1 (Feitsma et al. 2022); Leukocyte‐associated immunoglobulin‐like receptor (LAIR1), 3KGR_1 (Brondijk et al. 2010); Integrin alpha‐2 (ITGA2), 1DZI_1 (Emsley et al. 2000); Discoidin domain‐containing receptor 2 (DDR2), 2WUH (Carafoli et al. 2009); Megakaryocyte and platelet inhibitory receptor G6b (MPIG6B), CBP: 6R0X (Vögtle et al. 2019); C‐type mannose receptor 2 (MRC2), 5AO5_1 (Paracuellos et al. 2015); Mannan‐binding lectin serine protease 1 (MASP1), 3DEM (Nan et al. 2017); Complement C1s subcomponent (C1S), 4LOR_1 (Girija et al. 2013); Serine proteinase inhibitors (SERPINH1), 4AU2 (Widmer et al. 2012); Secreted protein acidic and rich in cysteine (SPARC), 2V53_1 (Hohenester et al. 2008); von Willebrand factor (vWF), 1ATZ_1 (Brondijk et al. 2012); Matrix metalloproteinase‐1 (MMP‐1), 4AUO (Manka et al. 2012); Kelch repeat and BTB domain‐containing protein 4 (KBTBD4), PDB structure not present, Uniprot ID: Q9NVX7; Fibronectin (I), FN1, 3EJH_1 (Erat et al. 2009). For two entries, ITGA11 and DDR1, the CBDs were determined by similarity via BlastP from ITGA2 and DDR2, respectively.

The structural model of At5‐MMP was carried out by using the program SWISS‐MODEL (https://swissmodel.expasy.org/, Waterhouse et al. 2018). The template used was the CBD of HsMMP1 [PDB accession code: 4AUO.1pdb modifying A200E to match the residue present in the active site (Chung et al. 2004)]. AtSERPIN1 was modeled on the canine SERPINH1 protein (PDB accession code: 3ZHA), which is almost identical (99%) to the human HsSERPINH1 (alignment not shown) and, in this case, was co‐crystallized with the collagen triple helix and therefore more informative.

The list of Uniprot IDs and the GRAMENE database accession for the *Solanum lycopersicum* orthologs presented in Table 2 was retrieved by the TAIR database accession when present; alternatively, they were identified by the UniProt BLAST function employing *Arabidopsis thaliana* identified proteins as queries.

## 3 Results and Discussion

**FIGURE 1:**
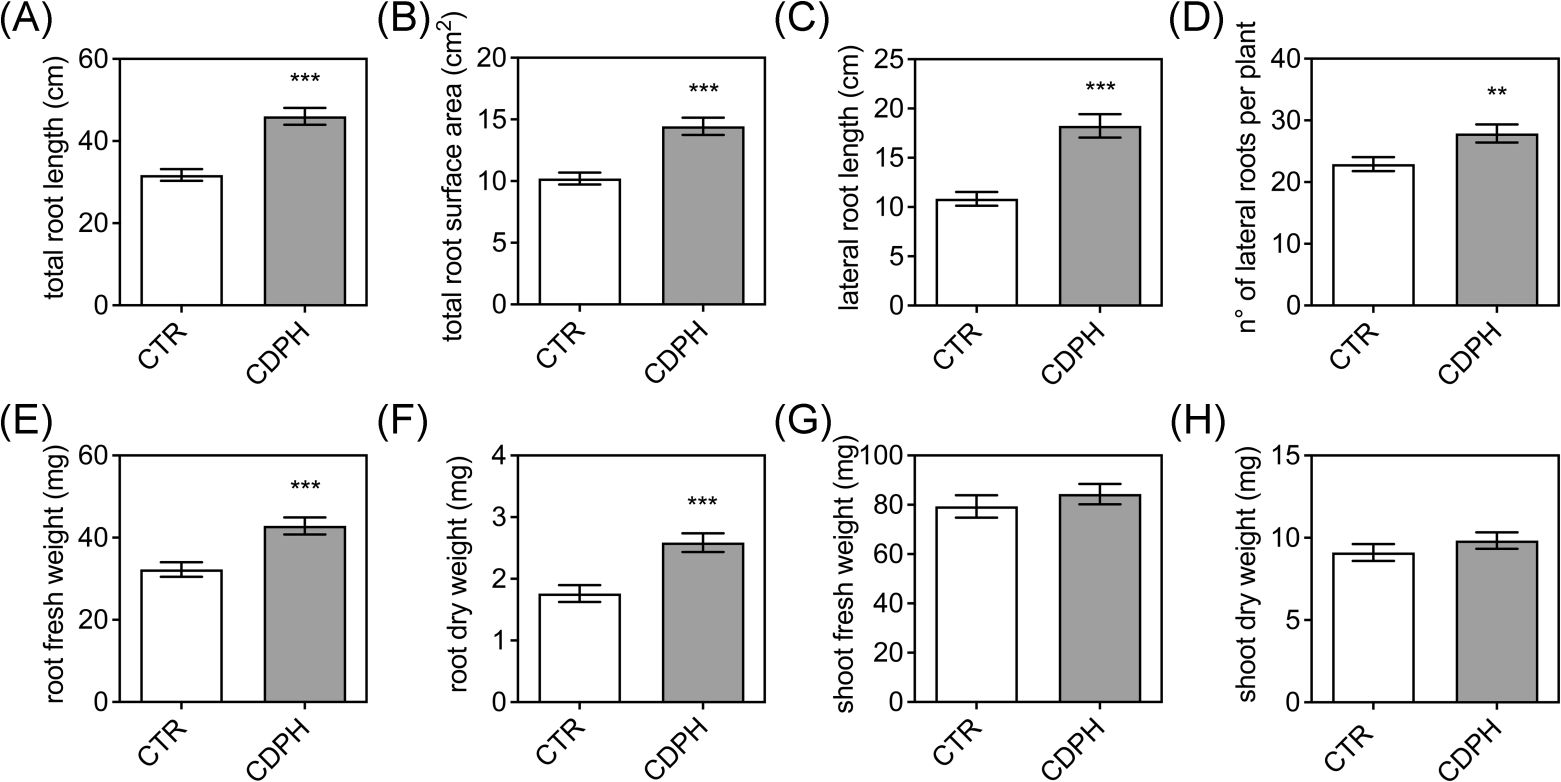
*Solanum lycopersicum* root and shoot parameters. (A) Total root length, (B) total surface area, (C) lateral root length (D) number of lateral roots, (E) root fresh weight, (F) dry weight, (G) shoot fresh weight and (H) shoot dry weight after 7 days of hydroponic growth with the addition of either inorganic N (CTR) or of the CDPH. Data, derived from three independent experiments, are expressed as mean ± S.E.M. (*n* = 76). Statistical method: Two‐tailed Welch's *t*‐test (*p* < 0.05).

The treatment with CDPH significantly promoted root growth in *Solanum lycopersicum* seedlings (Figure 1). Treated seedlings developed roots that were 45% longer and 41% wider than those of control plants (Figure 1A,B), with a 68% increase in lateral root length (Figure 1C) and a higher number of lateral roots (Figure 1D). Overall, the root system of treated plants exhibited greater biomass than control plants, with fresh and dry root weights increasing by 33% and 47%, respectively. In contrast, shoot biomass was not significantly affected by CDPH application (Figure 1G,H). The stimulatory effect of CDPH on root growth was previously observed in maize, both in optimal conditions and under abiotic stress (hypoxia and water stress) (Santi et al. 2017; Ambrosini et al. 2021).

In silico sequence analyses were conducted to find human CBPs (collagen‐binding proteins), and 17 entries were identified (Table S1). The list included three LRCs (OSCAR, GP6, LAIR1), two alpha integrins (ITGA2 and ITGA11), two DDRs (DDR1 and DDR2), MPIG6B, MRC2, MASP1, C1S, SERPINH1, SPARC, vWF, MMP‐1, KBTBD4, and FN1.

To improve the homology search, the CBDs (collagen‐binding domains) of the CBPs in the list were employed for the analysis when available. For MPIG6B, SERPINH1, and KBTBD4, a CBD was not found or was not clearly established; therefore, the whole protein sequence was used for the following analysis. The identified CBDs or CBPs were then used for an extensive homology search on *Arabidopsis thaliana* using the state‐of‐the‐art HHpred prediction server (Meier and Söding 2015). We focused on *Arabidopsis thaliana* because its protein databases are significantly more comprehensive and well‐annotated compared to those of other plant species. This allowed for a more reliable identification of putative CBPs and facilitated structural and functional predictions, providing a solid foundation for future validation in other plant models such as *Solanum lycopersicum* . Overall, 358 entries with an *e* value ≤ 0.001 were retrieved, heterogeneously distributed among the list of human CBPs (Table S2). The search with MPIG6B, MASP1, C1S, KBTBD4, FN1, and with the three LRCs did not reveal any statistically significant homologous protein. DDR1 and DDR2 both displayed the same plant homologue, the alpha‐L‐fucosidase FUC1, with slightly different e‐values (for DDR2 above the threshold). The MRC2 was also connected only to one plant homologue, a C‐type lectin domain‐containing protein. The entries that showed the higher number of outcomes were the CBP SERPINH1 (18 hits) and the CBDs MMP‐1, ITGA2, vWF, ITGA11, and SPARC (4, 63, 68, 70 and 133 hits respectively). After removing redundant entries, the final dataset included 213 proteins (Table S3), most of which (124 non‐redundant/161 total hits) were associated with calcium‐binding functions. These were grouped into four categories: calmodulin‐like proteins, copines, Ca‐binding EF‐hand proteins, and other Ca‐related proteins. Other groups represented included 18 Sec23/Sec24 transport proteins, 16 SERPINs (serine protease inhibitors), 13 Zinc finger proteins (C3HC4‐type RING finger), 7 RING proteins (domain ligase 2), and 4 matrixins (Table S3). For further analysis, we focused on 10 selected hits, ensuring that at least one plant homolog was represented for each of the eight human entries. Selection was based on biological criteria, requiring that the proteins are expressed in specific organs and cellular localizations where interactions with collagen‐derived oligopeptides are likely to occur. Based on the literature and the TAIR database, we focused on proteins expressed in roots and leaves. Both our data and previous studies (Ertani et al. 2009; Santi et al. 2017; Ambrosini et al. 2021) involving the addition of protein hydrolysates (PHs) to the nutrient solution have shown that roots are highly responsive to treatment. Additionally, evidence suggests that animal‐derived PHs can also exert biostimulant effects when applied as a foliar spray (Tejada et al. 2018). Concerning cellular localization, we selected proteins expressed in the extracellular matrix (ECM) or in the cytosol. In Table 1, we show the 10 proteins that have an identity percentage above 10% and that match the chosen biological.

**TABLE 1:** List of putative plant orthologues for CBDs and CBPs. *Arabidopsis thaliana* proteins found by homology via HHPred using as a query CBDs (ITGA2, ITGA11, DDR1, DDR2, OSCAR, GP6, LAIR, MRC, MASP, C1S, SPARC, vWF, MMP‐1, FN1) or CBPs (MPIG6B, SERPINH1, KBTBD4).

Reference CBD/CBP	HHPred predicted homologues in *Arabidopsis thaliana*	Tissue expression	Subcellular localization	Identity
**MMP‐1**	At5‐MMP, matrixin metalloproteinase	Roots, leaves, stem (to a lower extent in flowers) (Maidment et al. 1999)	Cell membrane (lipid‐anchor) (Maidment et al. 1999)	44%
**SERPINH**	AtSERPIN1, serine protease inhibitor	Ubiquitary (Lampl et al. 2013)	Cytosol (Lampl et al. 2013; Asqui et al. 2018)	22%
**SPARC**	AtCP1, Ca^2+^‐binding protein 1	Roots and flowers (Jang et al. 1998)	Cytosol (Jang et al. 1998)	20%
**MRC**	C‐type LecRLK, lectin receptor‐like kinase	Ubiquitary*	Cell membrane (single‐pass type I membrane protein)	19%
ITGA2, ITGA11, **vWF**	AtMUP24.2	Ubiquitary*	Plasma membrane**	18%
ITGA2, ITGA11, **vWF**	AtSEC24A	Mainly expressed in pollen, leaves, inflorescences, roots and stems, and, to a lower extent, in cotyledons, petioles and hypocotyls (Sato and Nakano 2007)	Cytoplasmic vesicle COPII, ER membrane, cytosol (Sato and Nakano 2007)	17%
**ITGA2**, vWF	Inter‐alpha‐trypsin inhibitor heavy chain‐like protein	Ubiquitary*	Plasma membrane**	17%
**DDR1**	AtFUC1, alpha‐L‐fucosidase 1	Ubiquitary*	ECM (de la Torre et al. 2002)	14%
ITGA2, ITGA11, **vWF**	AtRPN10, 26S proteasome non‐ATPase regulatory subunit 4 homolog	Ubiquitous, highest expression in flowers (Marshall et al. 2019)	Nucleus, plasma membrane, proteasome complex (Marshall et al. 2019)	10%
**ITGA2**, ITGA11, vWF	AtBABAM1, BRISC and BRCA1‐A complex member 1	Ubiquitary*	Cytosol, nucleus**	10%

*Note:* Only hits with an *e* value ≤ 0.001 were considered. When a plant protein was retrieved from multiple queries, then the identity percentage refers to the hit with a higher percentage of identity (and it is indicated in bold). Asterisks indicate that the tissue expression or subcellular localization were retrieved respectively from the database TAIR (*) and UniProt (**), while for the other entries we reported information present in the literature.

The data obtained for *Arabidopsis thaliana* were used to identify homologous proteins in *Solanum lycopersicum* (Table 2). These results suggest that homology can be established between mammalian and plant proteins. Since HsMMP‐1 and HsSERPINH1 in mammals and their homologues At5‐MMP and AtSERPIN1 in plants showed the highest identity percentages among the retrieved proteins, we chose to focus our discussion on these pairs. HsMMP‐1 is a matrix metalloproteinase (zinc‐containing endopeptidase) secreted in the ECM by different cell types (i.e., fibroblasts, keratinocytes, endothelial cells, macrophages and hepatocytes) and it can recognize and cleave collagen I, II, III, VII, VIII, X, gelatin (a mixture of peptides derived from the disruption of fibrillar collagen cross‐linkages between the polypeptide chains and a partial breakage of polypeptide bonds) as well as aggrecan, versican, perlecan, casein, nidogen, serpins, and tenascin‐C (McCawley and Matrisian 2001; Chang 2023). The *HsMMP‐1* gene encodes for a 469 AAs protein that comprises a signal peptide, a prodomain, a catalytic domain (where collagen fibrils are cleaved), and a hemopexin‐like C‐terminal domain (crucial to unwind the fibrillar structure exposing the cleavage site to the catalytic domain) (Bertini et al. 2012). The interaction with the collagen triple helix involves both the hemopexin and the catalytic domains, is temperature‐dependent, and was confirmed by biochemical assays employing short collagen model peptides of 27 AAs (Manka et al. 2012).

**TABLE 2:** List of *Solanum lycopersicum* putative orthologs for CBDs and CBPs.

Reference CBD/CBP	*Arabidopsis thaliana*	*Solanum lycopersicum*
**MMP‐1**	At5‐MMP, matrixin metalloproteinase Q9ZUJ5, *At1g59970*	SlMMP1, matrix metalloproteinase I7JCM3, *Solyc04g005050*
**SERPINH**	AtSERPIN1, serine protease inhibitor A0A1P8API2, *At1g47710*	Serpin domain‐containing protein A0A3Q7GAG7, *Solyc04g079440.3*
**SPARC**	AtCP1, Ca^2+^‐binding protein 1 Q9FDX6, *At5g49480*	EF‐hand domain‐containing protein A0A3Q7EET5, *Solyc01g058720.3*
**MRC**	C‐type LecRLK, lectin receptor‐like kinase Q9C823, *At1g52310*	Protein kinase domain‐containing protein A0A3Q7J058, *Solyc02g068370.3.1*
ITGA2, ITGA11, **vWF**	AtMUP24.2 Q9FF49, *At5g60710*	Uncharacterized protein A0A3Q7J058, *Solyc11g069600.2*
ITGA2, ITGA11, **vWF**	AtSEC24A Q9SFU0, *At3g07100*	Protein transport protein Sec24‐like A0A3Q7F833, *Solyc02g082220.3*
**ITGA2**, vWF	Inter‐alpha‐trypsin inhibitor heavy chain‐like protein A0A1P8ANU9, *At1g72500*	VWFA domain‐containing protein A0A3Q7FW48, *Solyc03g122270.3*
**DDR1**	AtFUC1, alpha‐L‐fucosidase 1 Q8GW72, *At2g28100*	Alpha‐L‐fucosidase A0A3Q7J033, *Solyc11g069000.2.1*
ITGA2, ITGA11, **vWF**	AtRPN10, 26S proteasome non‐ATPase regulatory subunit 4 homolog P55034, *At4g38630*	26S proteasome regulatory subunit RPN10 A0A3Q7F6N7, *Solyc02g083710.3*
**ITGA2**, ITGA11, vWF	AtBABAM1, BRISC and BRCA1‐A complex member 1 O82638, *At4g32960*	BRISC and BRCA1‐A complex member 1 A0A3Q7JEC8, *Solyc12g095920.2*

*Note:* For each protein, the UniProt ID is listed. For *Arabidopsis thaliana* , the gene ID refers to the TAIR database accession, while for *Solanum lycopersicum* , it refers to the Gramene database accession. *Solanum lycopersicum* orthologs were retrieved by the TAIR database accession when present; alternatively, they were identified by the UniProt BLAST function employing *Arabidopsis thaliana* identified proteins as queries.

The *Arabidopsis thaliana* homologue of HsMMP‐1, At5‐MMP, encodes a zinc‐dependent endopeptidase (Maidment et al. 1999). At5‐MMP is one of the MMPs identified in *Arabidopsis thaliana* , which function as active proteases with potentially overlapping but distinct roles in extracellular matrix (ECM) remodeling, degradation, and/or shedding of its components (Marino et al. 2014). These proteins share a basic structural organization, consisting of a signal peptide, a propeptide domain, and a catalytic domain (Flinn 2008). As with other plant MMPs, all five *Arabidopsis thaliana* MMPs lack a hemopexin‐like domain (Massova et al. 1998). However, in four of these proteins (AtMMP1, AtMMP2, AtMMP3, and AtMMP5), a putative C‐terminal transmembrane domain has been predicted (Maidment et al. 1999; Flinn 2008). For AtMMP2, AtMMP4, and AtMMP5, a glycosylphosphatidylinositol (GPI)‐anchored site has been predicted (Flinn 2008). At5‐MMP is constitutively expressed in leaves, buds, and siliques, with the highest expression levels observed in roots (Mishra et al. 2021). Functional characterization of the At5‐MMP knock‐out mutant (*At5mmpKO*) revealed reduced primary root growth, fewer lateral roots, increased stomatal conductance, and decreased water‐use efficiency under standard growth conditions compared to WT plants. Additionally, the mutants displayed impaired root‐to‐shoot auxin transport and reduced abscisic acid (ABA) accumulation in roots. Upon NaCl treatment, *At5mmpKO* plants exhibited heightened sensitivity to osmotic stress and an alteration in the composition of the rhizosphere bacterial community relative to WT (Mishra et al. 2021). *At5‐MMP* is involved in plant responses to biotic stress (Zhao et al. 2017). These authors reported that *At5‐MMP* expression is transiently induced at 16 h post‐inoculation (hpi) in *Arabidopsis thaliana* leaves following infection with *Botrytis cinerea*. Additionally, homozygous *at5‐mmp* mutants exhibited increased susceptibility to the fungus compared to wild‐type plants. Interestingly, the homologous *Solanum lycopersicum* protein encoded by *Sl3‐MMP* (*Solyc04g005050*), one of the five MMP genes identified in *Solanum lycopersicum* , has also been implicated in resistance to *Botrytis cinerea* and *Pseudomonas syringae* pv. tomato (Li et al. 2015). Given that At5‐MMP and its tomato homolog Sl3‐MMP are both implicated in responses to biotic stresses, particularly *Botrytis cinerea*, we propose that tomato MMPs may share functional roles with their *Arabidopsis thaliana* counterparts. In our study, these functions appear to be activated by CDPH application. Future investigations in tomato could clarify whether Sl3‐MMP contributes to collagen‐derived peptide perception, potentially linking this interaction to the enhanced root development we observed (Figure 1).

**FIGURE 2:**
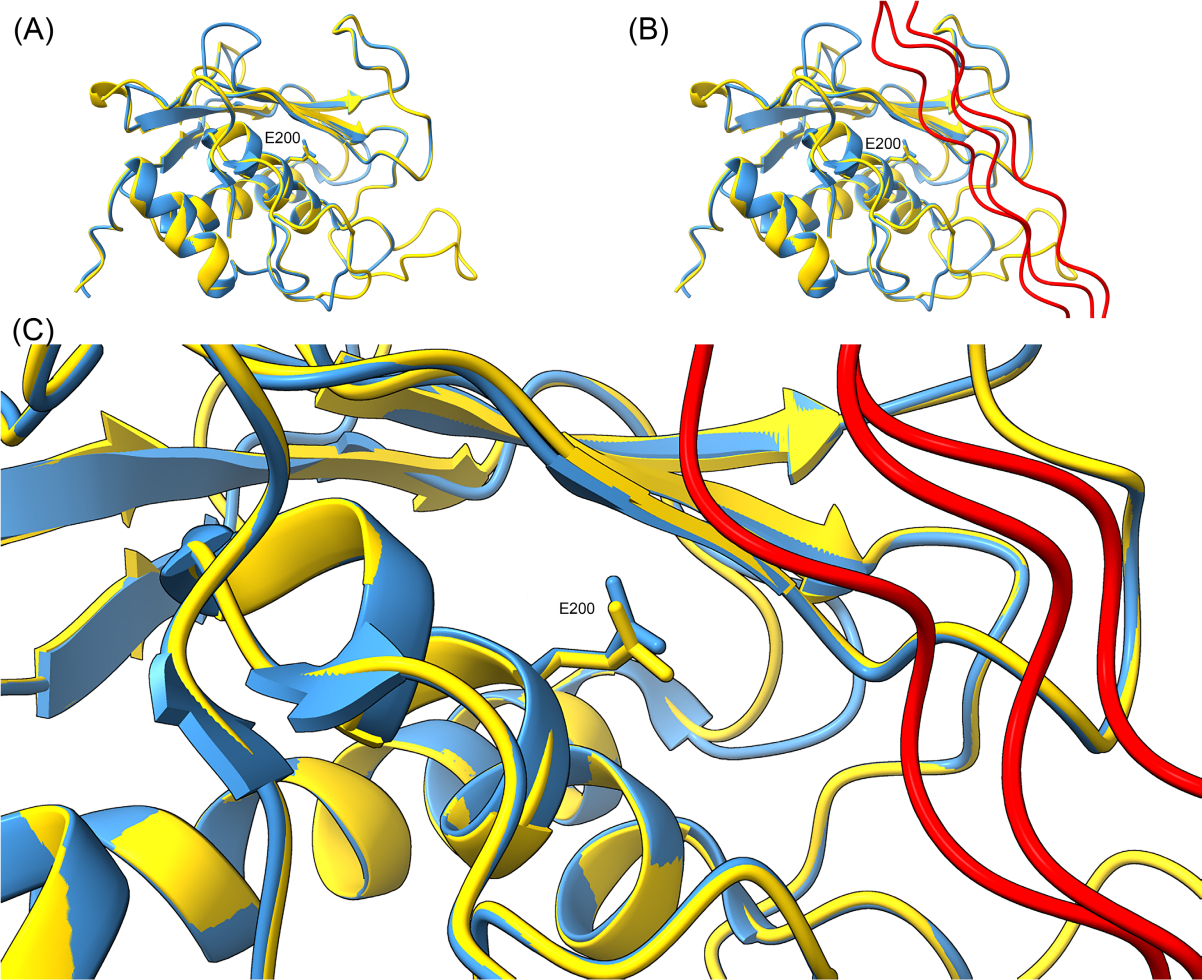
Structural alignment of At5‐MMP and HsMMP‐1 CBD. Ribbon structure of At5‐MMP (gold) modeled over the CBD of HsMMP‐1 (royal blue) in (A) the absence or (B) presence of a collagen fibril (red). The picture in (B) is zoomed in (C). HsMMP‐1 residue E200 is shown as a stick structure and clearly labeled in all figures. Zinc atoms are displayed in maroon. The image was created with UCSF ChimeraX: HsMMP‐1, F81 to C259 (PDB 4AUO); At5‐MMP Y143 to G319.

Despite increasing evidence of the roles played by plant MMPs in development and stress responses, their natural substrates remain largely unknown. In contrast to animal systems, where MMPs predominantly target ECM proteins (Pardo and Selman 2005), the identity of plant MMP substrates has yet to be clearly established. Plant MMPs exhibited in vitro proteolytic activity toward bovine myelin basic protein, gelatin, and synthetic peptides containing the scissile Gly‐Leu/Ile bond of collagen (McGeehan et al. 1992; Maidment et al. 1999; Delorme et al. 2000). This raises the intriguing possibility that plant MMPs may target atypical ECM‐like substrates, including collagen or collagen‐like glycoproteins introduced or produced in specific physiological or experimental contexts. To explore this hypothesis further, we performed a structural modeling analysis comparing At5‐MMP with human MMP‐1 (HsMMP‐1), a well‐characterized collagenase (Figure 2). The CBD of HsMMP‐1, spanning residues F81 to C259, was used as a backbone to model the corresponding region of At5‐MMP (Y143 to G319). Strikingly, the residues predicted to interact with the collagen fibril show a high degree of structural conservation between the two proteins. Most notably, the glutamic acid residue E200, which is critical for collagenolytic activity in HsMMP‐1 (Chung et al. 2004; Gorantla et al. 2024), is conserved in all five *Arabidopsis thaliana* MMP homologues. In At5‐MMP, the equivalent residue is E271. We further extended our homology modeling analysis to the *Solanum lycopersicum* homolog Sl3‐MMP. The key glutamic acid residue (E200 in HsMMP‐1) is also conserved in Sl3‐MMP. The resulting *Solanum lycopersicum* protein model, generated using the catalytic binding domain (CBD) of HsMMP‐1 as a structural template, showed a sequence identity of 47.27%.

**FIGURE 3:**
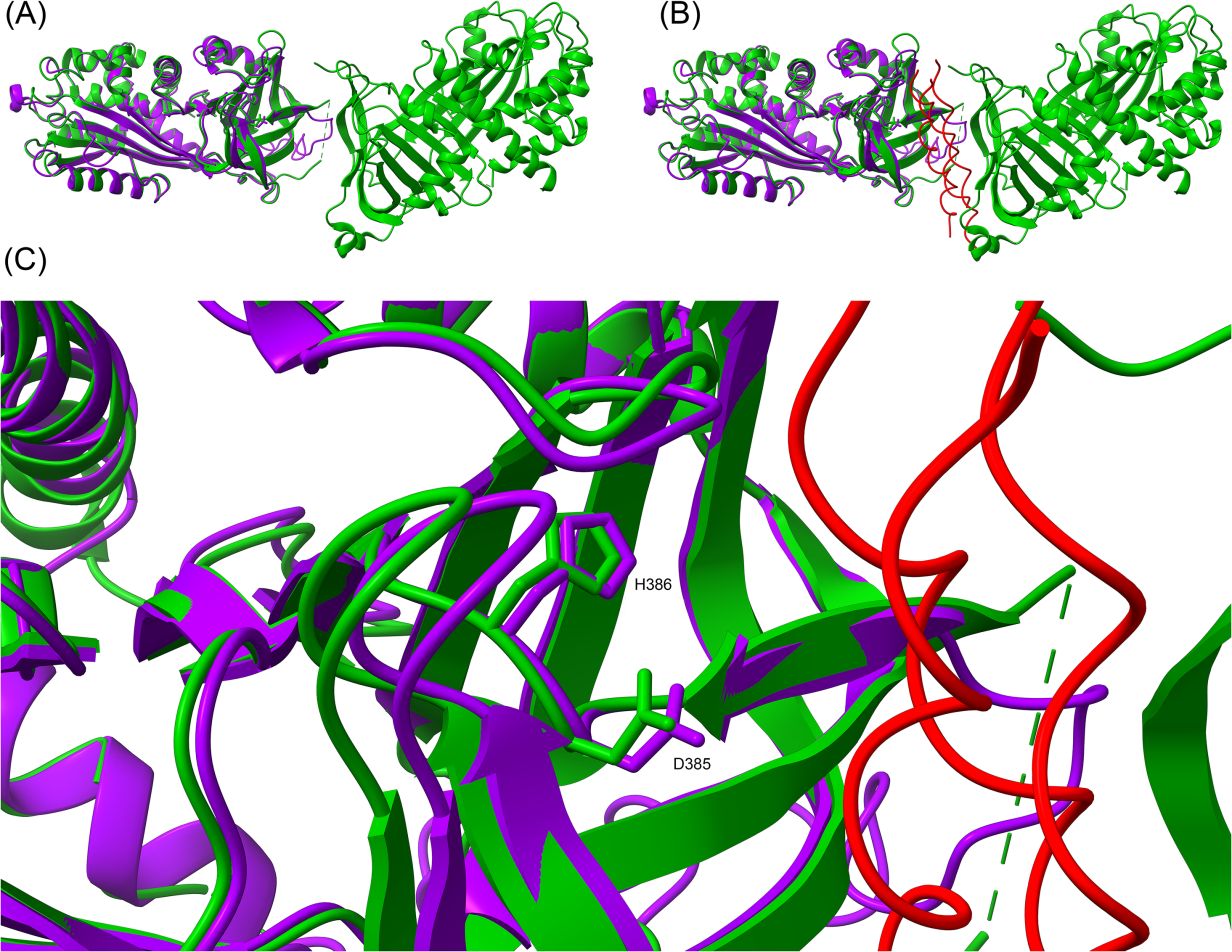
Structural alignment of AtSERPIN1 and HsSERPINH1 homodimer. Ribbon structure of AtSERPIN1 (violet) modeled over HsSERPINH1 (green) in (A) the absence or (B) presence of a collagen fibril (red). The picture in (B) is zoomed in (C). HsSERPINH1 residues D385 and H386 are shown as stick structures in all figures and clearly labeled in (C). The image was created with UCSF ChimeraX: HsSERPINH1, Chains Q and P, M35 to H425 (PDB 3ZHA); AtSERPIN1 V30 to H418.

In addition to the plant MMPs, our comparative analysis highlighted another protein of interest: a plant serpin (AtSERPIN1), which displayed the second‐highest sequence identity (22%) to its human counterparts (HsSERPINH1). HsSERPINH1, also known as Hsp47 or CBP1, is a molecular chaperone localized in the lumen of the endoplasmic reticulum (ER) and is crucial for collagen maturation and secretion (Widmer et al. 2012) since it inhibits procollagen local unfolding and aggregation (Ito and Nagata 2019). While the mammal protein harbors an RDEL signaling peptide at the C‐terminus, which allows its return to the ER via the KDEL receptor (Widmer et al. 2012), the plant homolog does not possess such a sequence. Another difference between the two serpins is that AtSERPIN1 functions as an inhibitor of proteases, as most of the serpins in all kingdoms do, while HsSERPINH1 is a molecular chaperone that does not possess serine protease inhibitory activity (Hirayoshi et al. 1991). AtSERPIN1 is the best‐characterized plant serpin among the eight encoded by the *Arabidopsis thaliana* genome (Fluhr et al. 2012). It plays a key role in plant stress responses by interacting with various endogenous proteases. Notably, AtSERPIN1 can inactivate the cysteine protease Responsive to Dessication 21 (RD21), thereby modulating pathogen‐induced programmed cell death (Shindo et al. 2012; Lampl et al. 2013). Besides, AtSERPIN1 inhibits the metacaspase AtMC1, a positive regulator of hypersensitive response‐associated cell death (Asqui et al. 2018). To date, no functional characterization of serpin proteins has been reported in *Solanum lycopersicum* . Regarding the interaction with collagen, numerous studies have identified key residues involved in binding by the human serpin HsSERPINH1, whereas no such information is currently available for its plant homolog. HsSERPINH1 has been shown to bind various collagen model peptides—even those as short as 15 amino acids—provided they form a triple helix, with binding stabilized by salt bridges and hydrophobic interactions (Ono et al. 2012; Widmer et al. 2012). Notably, two residues critical for collagen binding in HsSERPINH1, D385 and H386, are conserved in the plant serpin AtSERPIN1, suggesting a possible evolutionary conservation of binding functionality (Widmer et al. 2012) (Figure 3).

## 4 Conclusions

In conclusion, this study offers a pioneering molecular perspective on the interaction between plant proteins and collagen‐derived peptides used in biostimulant formulations. Through an in silico approach, we identified putative plant targets—including chaperones and protease inhibitors—that are closely associated with root development and with plant responses to both abiotic and biotic stresses. These molecular interactions align with the physiological effects previously observed upon application of the collagen‐derived biostimulant. Notably, since the collagen‐derived peptides retain the polyproline II (PPII) helical conformation of native collagen (Ambrosini et al. 2022)—a structural motif also found in plant cell wall extensins (van Holst and Varner 1984; Shpak et al. 2001; Herger et al. 2019)—we hypothesize that this conformation may be specifically recognized by plant proteins such as MMPs and SERPINs. This structural mimicry could underlie the observed interactions and partially explain the biostimulant's ability to modulate stress‐related pathways. Overall, our findings provide novel insights into the potential mechanisms of action of collagen‐derived biostimulants and establish a framework for future functional studies. Functional validation using *Arabidopsis thaliana* mutants for *At5‐MMP* and *AtSERPIN1* represents a logical next step to confirm our hypotheses. Although such analyses are beyond the scope of this Short Communication, they are currently in progress and will help identify the molecular components mediating plant–collagen peptide interactions. These studies are expected to clarify the molecular basis of CDPH activity and support the design of more targeted and effective biostimulant formulations for sustainable agricultural applications.

## Author Contributions

Z.V., A.Z., and S.A. conceived and designed the research. S.A. and A.G. directed the experiments. S.A. and M.P. performed the experiments. S.A., A.G., M.P., and T.P. analyzed and interpreted the data. S.A. and A.G. wrote the manuscript. Z.V., A.Z., and T.P. revised the manuscript. All authors contributed to the article and approved the submitted version.

## Supporting information


**Table S1:** List of human CBPs retrieved by *in silico* sequence analyses. Gene name(s), protein name, length in AA, identification of the CBD domain and its respective first and last AA were reported.
**Table S2:** Full list of putative plant orthologues for CBDs and CBPs. *Arabidopsis thaliana* proteins found by homology via HHPred using as a query CBDs (ITGA2, ITGA11, DDR1, DDR2, OSCAR, GP6, LAIR, MRC, MASP, C1S, SPARC, vWF, MMP‐1, FN1) or CBPs (MPIG6B, SERPINH1, KBTBD4). Only hits with an *e*value ≤ 0.001 were considered. Ref prot: human query protein employed for the HHPred search; Hit: ID from NCBI or PDB databases of the *Arabidopsis thaliana* proteins retrieved; Name: name of the proteins retrieved; Probability: probability that the hit is homologous to the query; *e*‐value: value indicating the expected number of false positives in a database search that would achieve a score equal to or better than that of this sequence match; Score: value indicating the total score obtained for an homology alignment; SS: partial score from the secondary structure comparison; Aligned cols: total number of matched columns in the query–template alignment; Target length: length in amino acids of the hit sequence; Group: protein group to which the hit protein belongs to (when identified).
**Table S3:** Non‐redundant list of putative plant orthologues for CBDs and CBPs. *Arabidopsis thaliana* proteins found by homology via HHPred using as a query CBDs (ITGA2, ITGA11, DDR1, DDR2, OSCAR, GP6, LAIR, MRC, MASP, C1S, SPARC, vWF, MMP‐1, FN1) or CBPs (MPIG6B, SERPINH1, KBTBD4). Only hits with an *e*‐value ≤ 0.001 were considered. Ref prot: human query protein employed for the HHPred search; Hit: ID from NCBI or PDB databases of the *Arabidopsis thaliana* proteins retrieved; Name: name of the proteins retrieved; Probability: probability that the hit is homologous to the query; *e*‐value: value indicating the expected number of false positives in a database search that would achieve a score equal to or better than that of this sequence match; Score: value indicating the total score obtained for an homology alignment; SS: partial score from the secondary structure comparison; Aligned cols: total number of matched columns in the query–template alignment; Target length: length in amino acids of the hit sequence; Group: protein group to which the hit protein belongs to (when identified).

## Data Availability

The data that support the findings of this study are available in the Supporting Information of this article.

## References

[ppl70512-bib-0001] Ambrosini, S. , B. Prinsi , A. Zamboni , et al. 2022. “Chemical Characterization of a Collagen‐Derived Protein Hydrolysate and Biostimulant Activity Assessment of Its Peptidic Components.” Journal of Agricultural and Food Chemistry 70, no. 36: 11201–11211. 36039940 10.1021/acs.jafc.2c04379 PMC9479078

[ppl70512-bib-0002] Ambrosini, S. , D. Sega , C. Santi , A. Zamboni , Z. Varanini , and T. Pandolfini . 2021. “Evaluation of the Potential Use of a Collagen‐Based Protein Hydrolysate as a Plant Multi‐Stress Protectant.” Frontiers in Plant Science 12: 600623. 33633760 10.3389/fpls.2021.600623 PMC7899969

[ppl70512-bib-0003] Asqui, S. L. , D. Vercammen , I. Serrano , et al. 2018. “AtSERPIN1 Is an Inhibitor of the Metacaspase AtMC1‐Mediated Cell Death and Autocatalytic Processing In Planta.” New Phytologist 218, no. 3: 1156–1166. 28157265 10.1111/nph.14446

[ppl70512-bib-0004] Bertini, I. , M. Fragai , C. Luchinat , et al. 2012. “Structural Basis for Matrix Metalloproteinase 1‐Catalyzed Collagenolysis.” Journal of the American Chemical Society 134, no. 4: 2100–2110. 22239621 10.1021/ja208338j PMC3298817

[ppl70512-bib-0005] Brondijk, T. H. C. , D. Bihan , R. W. Farndale , and E. G. Huizinga . 2012. “Implications for Collagen I Chain Registry From the Structure of the Collagen Von Willebrand Factor A3 Domain Complex.” Proceedings of the National Academy of Sciences of the United States of America 109, no. 14: 5253–5258. 22440751 10.1073/pnas.1112388109 PMC3325688

[ppl70512-bib-0006] Brondijk, T. H. C. , T. De Ruiter , J. Ballering , et al. 2010. “Crystal Structure and Collagen‐Binding Site of Immune Inhibitory Receptor LAIR‐1: Unexpected Implications for Collagen Binding by Platelet Receptor GPVI.” Blood 115, no. 7: 1364–1373. 20007810 10.1182/blood-2009-10-246322

[ppl70512-bib-0007] Carafoli, F. , D. Bihan , S. Stathopoulos , et al. 2009. “Crystallographic Insight Into Collagen Recognition by Discoidin Domain Receptor 2.” Structure 17, no. 12: 1573–1581. 20004161 10.1016/j.str.2009.10.012 PMC2807035

[ppl70512-bib-0008] Cavani, L. , C. Ciavatta , and C. Gessa . 2003. “Determination of Free L‐ and D‐Alanine in Hydrolysed Protein Fertilisers by Capillary Electrophoresis.” Journal of Chromatography A 985, no. 1‐2: 463–469. 12580515 10.1016/s0021-9673(02)01733-8

[ppl70512-bib-0009] Chang, M. 2023. “Matrix Metalloproteinase Profiling and Their Roles in Disease.” RSC Advances 13: 6304. 36825288 10.1039/d2ra07005g PMC9942564

[ppl70512-bib-0010] Chung, L. , D. Dinakarpandian , N. Yoshida , et al. 2004. “Collagenase Unwinds Triple‐Helical Collagen Prior to Peptide Bond Hydrolysis.” EMBO Journal 23, no. 15: 3020–3030. 15257288 10.1038/sj.emboj.7600318 PMC514933

[ppl70512-bib-0011] Cristiano, G. , E. Pallozzi , G. Conversa , V. Tufarelli , and B. De Lucia . 2018. “Effects of an Animal‐Derived Biostimulant on the Growth and Physiological Parameters of Potted Snapdragon (*Antirrhinum majus* L.).” Frontiers in Plant Science 9: 861. 29973949 10.3389/fpls.2018.00861 PMC6019948

[ppl70512-bib-0060] de la Torre, F. , J. Sampedro , I. Zarra , and G. Revilla . 2002. “AtFXG1, an Arabidopsis Gene Encoding α‐l‐Fucosidase Active against Fucosylated Xyloglucan Oligosaccharides.” Plant Physiology 128, no. 1: 247–255. 11788770 PMC148987

[ppl70512-bib-0053] Delorme, V. G. R. , P. F. McCabe , D. J. Kim , and C. J. Leaver . 2000. “A Matrix Metalloproteinase Gene Is Expressed at the Boundary of Senescence and Programmed Cell Death in Cucumber.” Plant Physiology 123, no. 3: 917–928. 10889240 10.1104/pp.123.3.917 PMC59054

[ppl70512-bib-0012] du Jardin, P. 2015. “Plant Biostimulants: Definition, Concept, Main Categories and Regulation.” Scientia Horticulturae 196: 3–14.

[ppl70512-bib-0013] Elango, J. , C. Hou , B. Bao , et al. 2022. “The Molecular Interaction of Collagen With Cell Receptors for Biological Function.” Polymers 14, no. 5: 876. 35267698 10.3390/polym14050876 PMC8912536

[ppl70512-bib-0014] Emsley, J. , C. G. Knight , R. W. Farndale , M. J. Barnes , and R. C. Liddington . 2000. “Structural Basis of Collagen Recognition by Integrin α2β1.” Cell 101, no. 1: 47–56. 10778855 10.1016/S0092-8674(00)80622-4

[ppl70512-bib-0015] Erat, M. C. , D. A. Slatter , E. D. Lowe , et al. 2009. “Identification and Structural Analysis of Type i Collagen Sites in Complex With Fibronectin Fragments.” Proceedings of the National Academy of Sciences of the United States of America 106, no. 11: 4195–4200. 19251642 10.1073/pnas.0812516106 PMC2649207

[ppl70512-bib-0016] Ertani, A. , L. Cavani , D. Pizzeghello , et al. 2009. “Biostimulant Activity of Two Protein Hydrolyzates in the Growth and Nitrogen Metabolism of Maize Seedlings.” Journal of Plant Nutrition and Soil Science 172, no. 2: 237–244.

[ppl70512-bib-0017] Ertani, A. , D. Pizzeghello , A. Altissimo , and S. Nardi . 2013. “Use of Meat Hydrolyzate Derived From Tanning Residues as Plant Biostimulant for Hydroponically Grown Maize.” Journal of Plant Nutrition and Soil Science 176, no. 2: 287–295.

[ppl70512-bib-0018] Feitsma, L. J. , H. C. Brondijk , G. E. Jarvis , et al. 2022. “Structural Insights Into Collagen Binding by Platelet Receptor Glycoprotein VI.” Blood 139, no. 20: 3087–3098. 35245360 10.1182/blood.2021013614

[ppl70512-bib-0048] Flinn, B. S. 2008. “Plant Extracellular Matrix Metalloproteinases.” Functional Plant Biology 35, no. 12: 1183–1193. 32688865 10.1071/FP08182

[ppl70512-bib-0062] Fluhr, R. , N. Lampl , and T. H. Roberts . 2012. “Serpin Protease Inhibitors in Plant Biology.” Physiologia Plantarum 145: 95–102. 22085334 10.1111/j.1399-3054.2011.01540.x

[ppl70512-bib-0054] Gorantla, K. R. , A. Krishnan , S. O. Waheed , et al. 2024. “Novel Insights into the Catalytic Mechanism of Collagenolysis by Zn(II)‐Dependent Matrix Metalloproteinase‐1.” Biochemistry 63, no. 15: 1925–1940. 38963231 10.1021/acs.biochem.4c00076 PMC11309001

[ppl70512-bib-0019] Girija, U. V. , A. R. Gingras , J. E. Marshall , et al. 2013. “Structural Basis of the C1q/C1s Interaction and Its Central Role in Assembly of the C1 Complex of Complement Activation.” Proceedings of the National Academy of Sciences of the United States of America 110, no. 34: 13916–13920. 23922389 10.1073/pnas.1311113110 PMC3752233

[ppl70512-bib-0020] Haywood, J. , J. Qi , C. C. Chen , et al. 2016. “Structural Basis of Collagen Recognition by Human Osteoclast‐Associated Receptor and Design of Osteoclastogenesis Inhibitors.” Proceedings of the National Academy of Sciences of the United States of America 113, no. 4: 1038–1043. 26744311 10.1073/pnas.1522572113 PMC4743793

[ppl70512-bib-0021] Herger, A. , K. Dünser , J. Kleine‐Vehn , and C. Ringli . 2019. “Leucine‐Rich Repeat Extensin Proteins and Their Role in Cell Wall Sensing.” Current Biology 29, no. 17: R851–R858. 31505187 10.1016/j.cub.2019.07.039

[ppl70512-bib-0022] Hirayoshi, K. , H. Kudo , H. Takechi , et al. 1991. “HSP47: A Tissue‐Specific, Transformation‐Sensitive, Collagen‐Binding Heat Shock Protein of Chicken Embryo Fibroblasts.” Molecular and Cellular Biology 11, no. 8: 4036–4044. 2072906 10.1128/mcb.11.8.4036-4044.1991 PMC361208

[ppl70512-bib-0023] Hohenester, E. , T. Sasaki , C. Giudici , R. W. Farndale , and H. P. Bächinger . 2008. “Structural Basis of Sequence‐Specific Collagen Recognition by SPARC.” Proceedings of the National Academy of Sciences of the United States of America 105, no. 47: 18273–18277. 19011090 10.1073/pnas.0808452105 PMC2587565

[ppl70512-bib-0024] Ito, S. , and K. Nagata . 2019. “Roles of the Endoplasmic Reticulum–Resident, Collagen‐Specific Molecular Chaperone Hsp47 in Vertebrate Cells and Human Disease.” Journal of Biological Chemistry 294, no. 6: 2133–2141. 30541925 10.1074/jbc.TM118.002812 PMC6369284

[ppl70512-bib-0058] Jang, H. J. , K. T. Pih , S. G. Kang , et al. 1998. “Molecular Cloning of a Novel Ca2+ Binding Protein That Is Induced by NaCl Stress.” Plant Molecular Biology 37: 839–847. 9678579 10.1023/a:1006043006211

[ppl70512-bib-0025] Jariwala, N. , M. Ozols , M. Bell , et al. 2022. “Matrikines as Mediators of Tissue Remodelling.” Advanced Drug Delivery Reviews 185: 114240. 35378216 10.1016/j.addr.2022.114240

[ppl70512-bib-0056] Lampl, N. , N. Alkan , O. Davydov , and R. Fluhr . 2013. “Set‐Point Control of RD21 Protease Activity by AtSerpin1 Controls Cell Death in Arabidopsis.” Plant Journal 74, no. 3: 498–510. 10.1111/tpj.12141 23398119

[ppl70512-bib-0026] Li, D. , H. Zhang , Q. Song , et al. 2015. “Tomato Sl3‐MMP, a Member of the Matrix Metalloproteinase Family, Is Required for Disease Resistance Against *Botrytis Cinerea* and *Pseudomonas syringae* Pv. Tomato DC3000.” BMC Plant Biology 15: 143. 26070456 10.1186/s12870-015-0536-z PMC4465618

[ppl70512-bib-0027] Malécange, M. , R. Sergheraert , B. Teulat , E. Mounier , J. Lothier , and S. Sakr . 2023. “Biostimulant Properties of Protein Hydrolysates: Recent Advances and Future Challenges.” International Journal of Molecular Sciences 24, no. 11: 9714. 37298664 10.3390/ijms24119714 PMC10253749

[ppl70512-bib-0046] Maidment, J. M. , D. Moore , G. P. Murphy , G. Murphy , and I. M. Clark . 1999. “Matrix Metalloproteinase Homologues from *Arabidopsis thaliana*: Expression and Activity.” Journal of Biological Chemistry 274: 34706–34710. 10574937 10.1074/jbc.274.49.34706

[ppl70512-bib-0028] Manka, S. W. , F. Carafoli , R. Visse , et al. 2012. “Structural Insights Into Triple‐Helical Collagen Cleavage by Matrix Metalloproteinase 1.” Proceedings of the National Academy of Sciences of the United States of America 109, no. 31: 12461–12466. 22761315 10.1073/pnas.1204991109 PMC3411981

[ppl70512-bib-0047] Marino, G. , P. F. Huesgen , U. Eckhard , C. M. Overall , W. P. Schröder , and C. Funk . 2014. “Family‐Wide Characterization of Matrix Metalloproteinases from *Arabidopsis thaliana* Reveals Their Distinct Proteolytic Activity and Cleavage Site Specificity.” Biochemical Journal 457, no. 2: 335–346. 24156403 10.1042/BJ20130196

[ppl70512-bib-0061] Marshall, R. S. , Z. Hua , S. Mali , F. Mcloughlin , and R. D. Vierstra . 2019. “ATG8‐Binding UIM Proteins Define a New Class of Autophagy Adaptors and Receptors.” Cell 177, no. 3: 766–781. 30955882 10.1016/j.cell.2019.02.009 PMC6810650

[ppl70512-bib-0049] Massova, I. , L. P. Kotra , R. Fridman , and S. Mobashery . 1998. “Matrix Metalloproteinases: Structures, Evolution, and Diversification.” FASEB Journal 12, no. 12: 1075–1095. 9737711

[ppl70512-bib-0029] McCawley, L. J. , and L. M. Matrisian . 2001. “Matrix Metalloproteinases: They're Not Just for Matrix Anymore!” Current Opinion in Cell Biology 13, no. 5: 534–540. 11544020 10.1016/s0955-0674(00)00248-9

[ppl70512-bib-0052] McGeehan, G. , W. Burkhart , R. Anderegg , J. D. Becherer , J. W. Gillikin , and J. S. Graham . 1992. “Sequencing and Characterization of the Soybean Leaf Metalloproteinase: Structural and Functional Similarity to the Matrix Metalloproteinase Family.” Plant Physiology 99, no. 3: 1179–1183. 16668986 10.1104/pp.99.3.1179 PMC1080600

[ppl70512-bib-0030] Meier, A. , and J. Söding . 2015. “Automatic Prediction of Protein 3D Structures by Probabilistic Multi‐Template Homology Modeling.” PLoS Computational Biology 11, no. 10: e1004343. 26496371 10.1371/journal.pcbi.1004343 PMC4619893

[ppl70512-bib-0031] Mienaltowski, M. J. , and D. E. Birk . 2014. “Structure, Physiology, and Biochemistry of Collagens.” In Progress in Heritable Soft Connective Tissue Diseases, edited by J. Halper , vol. 802, 5–29. Springer. 10.1007/978-94-007-7893-1_2 24443018

[ppl70512-bib-0032] Mishra, L. S. , S. Y. Kim , D. F. Caddell , D. Coleman‐Derr , and C. Funk . 2021. “Loss of Arabidopsis Matrix Metalloproteinase‐5 Affects Root Development and Root Bacterial Communities During Drought Stress.” Physiologia Plantarum 172, no. 2: 1045–1058. 33616955 10.1111/ppl.13299 PMC8247326

[ppl70512-bib-0033] Moreno‐Hernández, J. M. , M. Á. Mazorra‐Manzano , J. A. Salazar‐Leyva , and I. Benítez‐García . 2022. Protein Hydrolysates as Biostimulants of Plant Growth and Development, 141–175. Springer.

[ppl70512-bib-0034] Nan, R. , C. M. Furze , D. W. Wright , J. Gor , R. Wallis , and S. J. Perkins . 2017. “Flexibility in Mannan‐Binding Lectin‐Associated Serine Proteases‐1 and ‐2 Provides Insight on Lectin Pathway Activation.” Structure 25, no. 2: 364–375. 28111019 10.1016/j.str.2016.12.014 PMC5300068

[ppl70512-bib-0057] Ono, T. , T. Miyazaki , Y. Ishida , and Y., M. Uehata, and K. Nagata. 2012. “Direct In Vitro and In Vivo Evidence for Interaction Between Hsp47 Protein and Collagen Triple Helix.” Journal of Biological Chemistry 287, no. 9: 6810–6818. 22235129 10.1074/jbc.M111.280248 PMC3307285

[ppl70512-bib-0035] Paracuellos, P. , D. C. Briggs , F. Carafoli , T. Lončar , and E. Hohenester . 2015. “Insights Into Collagen Uptake by C‐Type Mannose Receptors From the Crystal Structure of Endo180 Domains 1‐4.” Structure 23, no. 11: 2133–2142. 26481812 10.1016/j.str.2015.09.004 PMC4635314

[ppl70512-bib-0051] Pardo, A. , and M. Selman . 2005. “MMP‐1: The Elder of the Family.” International Journal of Biochemistry & Cell Biology 37, no. 2: 283–288. 15474975 10.1016/j.biocel.2004.06.017

[ppl70512-bib-0036] Pituello, C. , S. Ambrosini , Z. Varanini , et al. 2022. Animal‐Derived Hydrolyzed Protein and Its Biostimulant Effects, 107–140. Springer.

[ppl70512-bib-0037] Ricard‐Blum, S. 2011. “The Collagen Family.” Cold Spring Harbor Perspectives in Biology 3, no. 1: a004978. 21421911 10.1101/cshperspect.a004978 PMC3003457

[ppl70512-bib-0038] Santi, C. , A. Zamboni , Z. Varanini , and T. Pandolfini . 2017. “Growth Stimulatory Effects and Genome‐Wide Transcriptional Changes Produced by Protein Hydrolysates in Maize Seedlings.” Frontiers in Plant Science 8: 433. 28424716 10.3389/fpls.2017.00433 PMC5371660

[ppl70512-bib-0059] Sato, K. , and A. Nakano . 2007. “Mechanisms of COPII Vesicle Formation and Protein Sorting.” FEBS Letters 581, no. 2076: 2082. 10.1016/j.febslet.2007.01.091 17316621

[ppl70512-bib-0055] Shindo, T. , J. C. Misas‐Villamil , A. C. Hörger , J. Song , and R. A. van der Hoorn . 2012. “A Role in Immunity for Arabidopsis Cysteine Protease RD21, the Ortholog of the Tomato Immune Protease C14.” PLoS One 7, no. 1: e29317. 22238602 10.1371/journal.pone.0029317 PMC3253073

[ppl70512-bib-0039] Shpak, E. , E. Barbar , J. F. Leykam , and M. J. Kieliszewski . 2001. “Contiguous Hydroxyproline Residues Direct Hydroxyproline Arabinosylation in *Nicotiana tabacum* .” Journal of Biological Chemistry 276, no. 14: 11272–11278. 11154705 10.1074/jbc.M011323200

[ppl70512-bib-0040] Tejada, M. , B. Rodríguez‐Morgado , P. Paneque , and J. Parrado . 2018. “Effects of Foliar Fertilization of a Biostimulant Obtained From Chicken Feathers on Maize Yield.” European Journal of Agronomy 96: 54–59.

[ppl70512-bib-0041] van Holst, G.‐J. , and J. E. Varner . 1984. “Reinforced Polyproline II Conformation in a Hydroxyproline‐Rich Cell Wall Glycoprotein From Carrot Root.” Plant Physiology 74, no. 2: 247–251. 16663405 10.1104/pp.74.2.247 PMC1066663

[ppl70512-bib-0042] Vögtle, T. , S. Sharma , J. Mori , et al. 2019. “Heparan Sulfates Are Critical Regulators of the Inhibitory Megakaryocyte‐Platelet Receptor G6B‐B.” eLife 8: e46840. 31436532 10.7554/eLife.46840 PMC6742478

[ppl70512-bib-0043] Waterhouse, A. , M. Bertoni , S. Bienert , et al. 2018. “SWISS‐MODEL: Homology Modelling of Protein Structures and Complexes.” Nucleic Acids Research 46: W296–W303. 29788355 10.1093/nar/gky427 PMC6030848

[ppl70512-bib-0044] Widmer, C. , J. M. Gebauer , E. Brunstein , et al. 2012. “Molecular Basis for the Action of the Collagen‐Specific Chaperone Hsp47/SERPINH1 and Its Structure‐Specific Client Recognition.” Proceedings of the National Academy of Sciences of the United States of America 109, no. 33: 13243–13247. 22847422 10.1073/pnas.1208072109 PMC3421173

[ppl70512-bib-0045] Wilson, H. T. , M. Amirkhani , and A. G. Taylor . 2018. “Evaluation of Gelatin as a Biostimulant Seed Treatment to Improve Plant Performance.” Frontiers in Plant Science 9: 1006. 30100911 10.3389/fpls.2018.01006 PMC6072858

[ppl70512-bib-0050] Zhao, P. , F. Zhang , D. Liu , J. Imani , G. Langen , and K. H. Kogel . 2017. “Matrix Metalloproteinases Operate Redundantly in Arabidopsis Immunity Against Necrotrophic and Biotrophic Fungal Pathogens.” PLoS One 12, no. 8: e0183577. 28832648 10.1371/journal.pone.0183577 PMC5568438

